# Integrated bioinformatics analysis of chromatin regulator EZH2 in regulating mRNA and lncRNA expression by ChIP sequencing and RNA sequencing

**DOI:** 10.18632/oncotarget.13169

**Published:** 2016-11-07

**Authors:** Yuan Li, Mei Luo, Xuejiao Shi, Zhiliang Lu, Shouguo Sun, Jianbing Huang, Zhaoli Chen, Jie He

**Affiliations:** ^1^ Department of Thoracic Surgery, National Cancer Center/Cancer Hospital, Chinese Academy of Medical Sciences and Peking Union Medical College, Beijing, People's Republic of China; ^2^ Central Laboratory, Beijing Luhe Hospital, Capital Medical University, Beijing, People's Republic of China

**Keywords:** EZH2, ChIP sequencing, RNA-sequencing, long noncoding RNA, cancer

## Abstract

Enhancer of zeste homolog 2 (EZH2), a dynamic chromatin regulator in cancer, represents a potential therapeutic target showing early signs of promise in clinical trials. EZH2 ChIP sequencing data in 19 cell lines and RNA sequencing data in ten cancer types were downloaded from GEO and TCGA, respectively. Integrated ChIP sequencing analysis and co-expressing analysis were conducted and both mRNA and long noncoding RNA (lncRNA) targets were detected. We detected a median of 4,672 mRNA targets and 4,024 lncRNA targets regulated by EZH2 in 19 cell lines. 20 mRNA targets and 27 lncRNA targets were found in all 19 cell lines. These mRNA targets were enriched in pathways in cancer, Hippo, Wnt, MAPK and PI3K-Akt pathways. Co-expression analysis confirmed numerous targets, mRNA genes (RRAS, TGFBR2, NUF2 and PRC1) and lncRNA genes (lncRNA LINC00261, DIO3OS, RP11-307C12.11 and RP11-98D18.9) were potential targets and were significantly correlated with EZH2. We predicted genome-wide potential targets and the role of EZH2 in regulating as a transcriptional suppressor or activator which could pave the way for mechanism studies and the targeted therapy of EZH2 in cancer.

## INTRODUCTION

Dynamic regulation of chromatin regulators at enhancers and promoters plays an important role in modulation of gene expression and cell fate determination [1, 2]. EZH2, the catalytic subunit of Polycomb repressive complex 2 (PRC2), methylates lysine 27 of histone H3 (H3K27) to promote transcriptional silencing [3]. Converging lines of investigation have implicated that EZH2 is likely to be an important mediator of tumor cell plasticity and involves in development and progression of serval cancers. The earliest and intensive study of a role for EZH2 in cancer was that EZH2 overexpression was associated with worse progression of prostate cancer [4]. Similar findings were also observed in other tumors such as breast cancer, bladder cancer, melanoma, as high level of EZH2 were shown to correlate with aggressiveness and advanced disease in each of these cancer types [5-7]. Given its role as a transcriptional regulator, researchers focus on the identification of EZH2 regulated target genes or pathways with great efforts. In prostate cancer and breast cancer, EZH2 has been shown to repress the expression of E-cadherin [8] and RUNX3 [9], resulted in promotion of EMT and invasive phenotype and increased cell proliferation, respectively. Moreover, independently, ectopic EZH2 expression has been found to confer a proliferative advantage upon noncancerous cells [5].

The canonical role of EZH2 is mainly to transcriptionally silence of tumor suppressor genes which depends on PRC2. However, several recent studies showed the non-canonical functions of EZH2, such as transcriptional activation of target genes, which are sometimes associated with malignant progression. In ER-positive breast cancer cells, EZH2 interacts with β-catenin and ER, and functionally enhances gens expression which is independent of PRC2 [10].

Long noncoding RNAs (lncRNAs), which are RNA transcripts longer than 200 nucleotides without coding capacity, are emerging as crucial regulators in tumorigenesis [11]. Growing evidence proved that lncRNAs involved in the epigenetic silencing of gene expression by EZH2. MALAT1 [12], DANCR [13], PVT1 [14] and lnc-beta-Catm [15] could recruit EZH2 and resulted in epigenetically silencing target genes expression. Moreover, EZH2 also interactively regulate lncRNAs expression, lncRNA SPRY4-IT1 [16] and lncRNA-LET [17] were repressed by EZH2.

These researches show the multifaceted role of EZH2 in human malignancies and challenges will be to understand not only the uniqueness in the context of different tumors, but also the complexity of roles of enzymatic activity as a transcriptional suppressor versus non-enzymatic activity as a transcriptional activator. However, current studies of EZH2 in cancer limited in hormonal tumors such as prostate cancer and breast cancer [18]. Regulation by EZH2 in other cancer types remains largely unstudied, especially for the lncRNA targets.

Based on the high throughput technology, such as ChIP sequencing and RNA sequencing, we can analyze the global changes in genome-wide DNA interaction sites and gene expression [19]. In this study, we highlighted the role of EZH2 in regulating mRNA and lncRNA expression by integrating ChIP sequencing analysis in 19 normal and cancer cell lines and co-expression analysis in ten cancer types from TCGA data. Our finding provided a global view on cell specific EZH2 transcription regulation and canonical and non-canonical roles of EZH2. These results revealed the involvement of EZH2 in transcriptional regulation of gene expression and provided potential targets for further research on EZH2.

## RESULTS

### EZH2 binding peaks in multiple normal and cancer cell lines and distribution features

**Figure 1:**
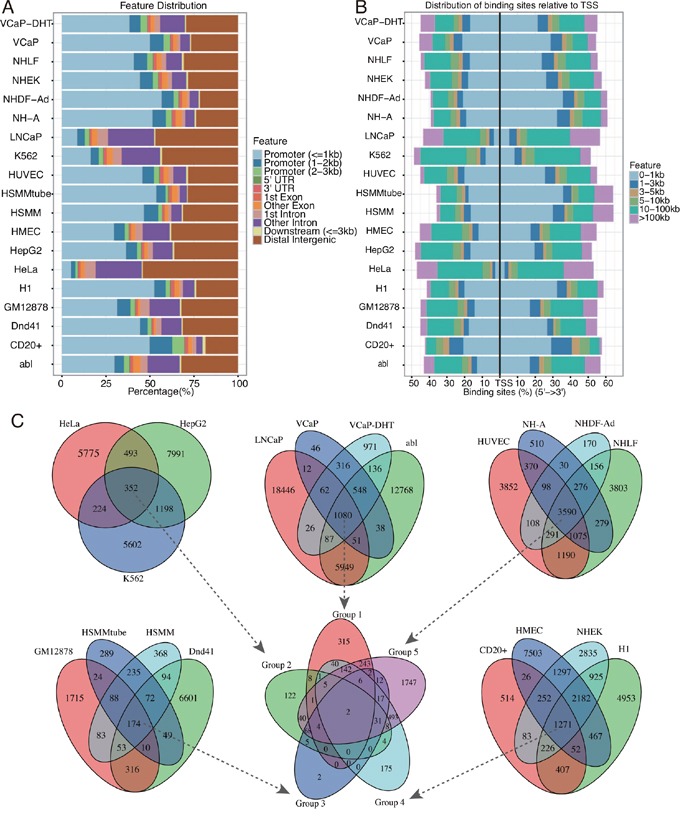
Characteristics of the binding peaks among the 19 cell lines **A.** Feature distribution of the binding peaks among the 19 cell lines. **B.** Distribution of binding sites relative to the TSS region. **C.** Venn diagram showed the overlapping binding peaks among the 19 cell lines. Peak intervals with a separation of maxgap of 1000bp or less were considered to be overlapped. 19 cell lines were separated into five groups and the overlapping binding peaks in each group were then further analyzed indicated by the arrows.

Based on MACS2 peak calling results, EZH2 binding peaks in different cell line ranged from 1,046 for HSMMtube to 28,588 for LNCaP with a median of 7,621 per cell line (Table 1). We analyzed distribution features of EZH2 binding sites on chromosome (Figure 1A and 1B). The results showed that EZH2 binding sites in most cell lines were centered on the promoter region (<= 3kb to TSS region) except LNCaP, K562 and HeLa cell lines which were centered on distal intergenic region (> 3kb to the TSS region) (Figure 1A). Among the EZH2 binding sites relative to promoter region, most were centered on 1kb region to the TSS region (Figure 1B), whereas binding sites in LNCaP, K562 and HeLa were located with 5-10 kb region to the TSS region (Figure 1B). Moreover, we also analyzed the overlapping of EZH2 binding peaks among different cell lines. Peak intervals with a separation of maxgap of 1,000bp or less were considered to be overlapped. 19 cell lines were divided into five groups. Cancer cell lines including LNCaP, abl, VCaP and VCaP-DHT were in group 1 while HeLa, HepG2 and K562 were in group 2. The other normal cell lines were divided into group 3-5 (Figure 1C). Venn diagram showed that there were hundreds to thousands of overlapping binding peaks in different group (Figure 1C). Further analysis showed that there was two overlapping binding peaks in all 19 cell lines (Figure 1C) one of which was located in the intron of coding gene COMMD3-BMI1 by annotation (Figure 3A).

**Table 1:** GSM number and cell lines information of the CHIP sequencing data

Data	Cell line	Cell type	Tissue	Peaks	mRNA Targets	lncRNA targets
GSM717404 [44]	VCaP	Epithelial	Prostate	2814	1397	1340
GSM717405 [44]	VCaP-DHT*	Epithelial	Prostate	3936	2241	2171
GSM1003470 [45]	CD20+	B lymphocyte	Blood	4741	1690	1497
GSM1003522 [45]	Dnd41	T lymphocyte	Blood	7621	5066	4306
GSM1003498 [45]	GM12878	Lymphocyte	Blood	2542	2062	1955
GSM1003524 [45]	H1	Embryonic stem	Embryo	11932	6684	5441
GSM1003501 [45]	HMEC	Epithelial	Mammary Gland	15215	7204	6284
GSM1003484 [45]	HSMM	Myoblast	Muscle	1318	880	854
GSM1003523 [45]	HSMMtube	Myoblast	Muscle	1046	737	703
GSM1003518 [45]	HUVEC	Epithelial	Vein	13196	6372	5189
GSM1003520 [45]	HeLa	Epithelial	Cervix	7058	3216	3178
GSM1003487 [45]	HepG2	Epithelial	Liver	10517	5751	4943
GSM1003576 [45]	K562	Erythroblast	Bone marrow	7588	4672	4024
GSM1003532 [45]	NH-A	Astrocytes	Brain	8072	4013	3444
GSM1003550 [45]	NHDF-Ad	Fibroblast	Skin	5859	3180	2770
GSM1003489 [45]	NHEK	Keratinocytes	Skin	11929	5226	4545
GSM1003529 [45]	NHLF	Fibroblast	Lung	14682	6290	5145
GSM969570 [46]	LNCaP	Epithelial	Prostate	28588	4950	5061
GSM969562 [46]	abl	Epithelial	prostate	25466	7255	6210

* VCaP-DHT was VCaP cell treated with 2 hours of 100nM dihydrotestosterone (DHT).

### Peak annotation, GO and KEGG enrichment analysis

**Figure 2:**
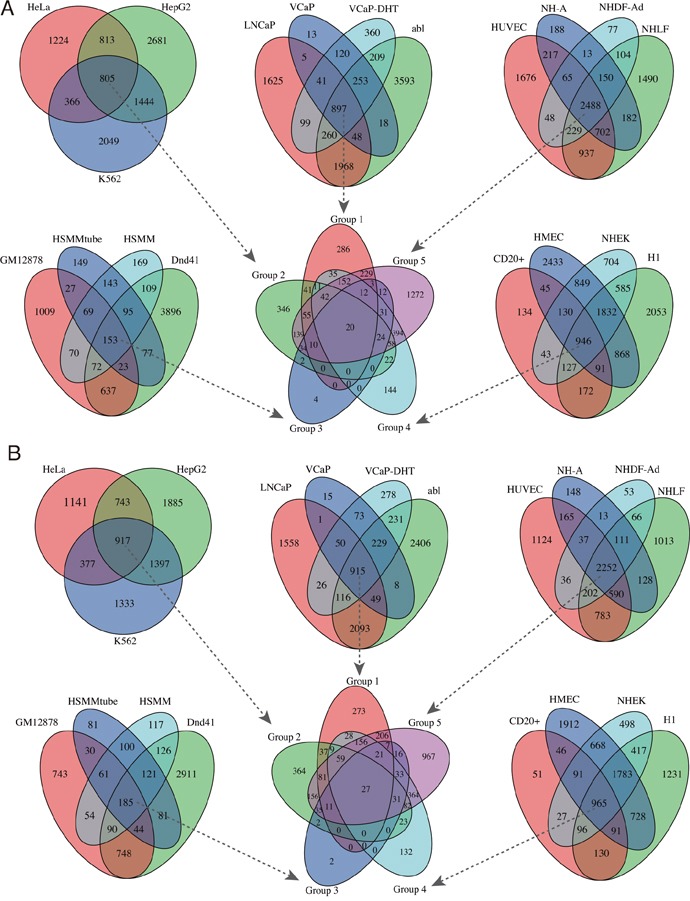
Venn diagram of the overlapping EZH2 target mRNAs and lncRNAs **A.** Venn diagram of EZH2 target mRNAs among the 19 cell lines. **B.** Venn diagram of EZH2 target lncRNAs among the 19 cell lines.

**Figure 3:**
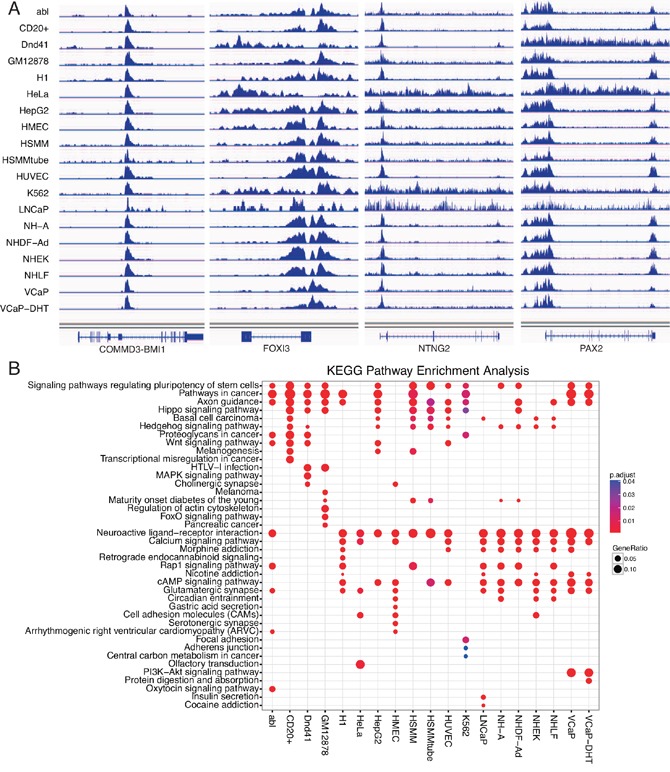
Binding of EZH2 at representative genomic loci and KEGG pathway enrichment of EZH2 target genes **A.** Binding of EZH2 at representative genomic loci. Binding peaks around or within shared target genes of COMMD3-BMI1, FOXI3, NTNG2 and PAX2 were presented in 19 cell lines. **B.** KEGG pathway analysis of EZH2 target genes in 19 cell lines.

Using ChIPseeker package, we detected the EZH2-regulated target genes around binding peaks. For mRNA targets, the identified target coding genes ranged from 737 in HSMMtube to 7,255 in abl cell with a median of 4,672 per cell line (Table 1 and Supplementary Table S1). For lncRNA targets, the identified target lncRNAs ranged from 703 in HSMMtube to 6,284 in HMEC cell with a median of 4,024 per cell line (Table 1 and Supplementary Table S2). Venn diagram showed that there were 20 overlapping mRNAs and 27 overlapping lncRNAs targets in all 19 cell lines (Figure 2). Notably, we also discovered a number of cancer or cell specific target mRNAs or lncRNAs. For instance, 286 target mRNAs and 273 target lncRNAs were cancer specific which were only enriched in the four prostate cancer cell lines. Binding of EZH2 at representative genomic loci were visualized using IGV (Figure 3A).

Combination with GO database, we conducted GO enrichment analysis for EZH2-regulated target genes across all the 19 cell lines (Supplementary Figure S1) (P<0.05). We discovered that EZH2 regulated the developmental process (biology process), plasma membrane (cellular component) and DNA binding (molecular function) in almost all the 19 cell lines (Supplementary Figure S1). For KEGG pathway enrichment analysis, signaling pathways regulation pluripotency of stem cells and pathways in cancer were significantly enriched in most of the cell lines while important pathways Hippo, Wnt, MAPK and PI3K-Akt signaling pathways were enriched in different cell lines (Figure 3B). Notably, PI3K-Akt signaling pathway were only enriched in two prostate cancer cell VCaP and VCaP-DHT (Figure 3B). In total, EZH2-regulated target genes showed shared similarities with differences of diverse characteristics and cell specificity.

### EZH2 binding patterns around promoter region

**Figure 4:**
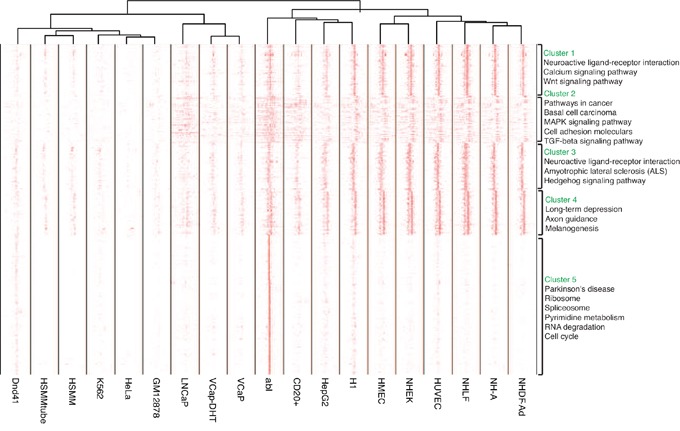
Heatmap of signal change of EZH2 binding sites within the promoters Promoters were defined as regions within 3kb of TSSs as default. 8,620 promoters were selected by excluding those where there were rare binding sites within. EZH2 binding sites could be divided into strong positioning signal and weak localization signal depending on ChIP sequencing signal intensity. Five clusters of promoters were generated and KEGG pathway enrichment analysis was performed.

To evaluate the binding patterns around the promoter region, we next systematically examined EZH2 binding sites across all promoters. 8,620 promoters were selected by excluding those where there were rare binding sites within. We then clustered the binding patterns based on their localization patterns across these loci and different cell lines. Figure 4 showed that the EZH2 binding sites could be divided into strong positioning signal and weak localization signal depending on ChIP sequencing signal intensity. The intensity of binding sites among these selected promoters varied from sample to sample and five clusters of promoters were generated (Figure 4). EZH2 binding sites in most of the cell lines showed signal within cluster 1-4 while only EZH2 binding sites in abl cell showed strong signal in cluster 5. Moreover, binding sites in cluster 2 were located in the flanking region of TSSs while binding sites in other clusters were mainly located in TSSs region. KEGG pathway enrichment analysis were also applied to the clusters separately. These results were largely consistent with KEGG pathway enrichment in Figure 3B. Signaling pathways associated with neuro development were enriched in all five clusters while important pathways such as Wnt, MAPK, TGF-beta and calcium signaling pathway were enriched in cluster 1 and 2. Pathways enriched in cluster 5 were ribosome, spliceosome, RNA degradation and cell cycle (Figure 3B).

### Co-expression analysis of EZH2 in TCGA data

Co-expression analysis of transcriptome data may provide interesting insights in understanding the gene and transcript level regulations in biological samples. In order to find out the potential targets of EZH2 and validation of ChIP sequencing data, co-expression analysis between EZH2 and other genes were applied.

**Figure 5:**
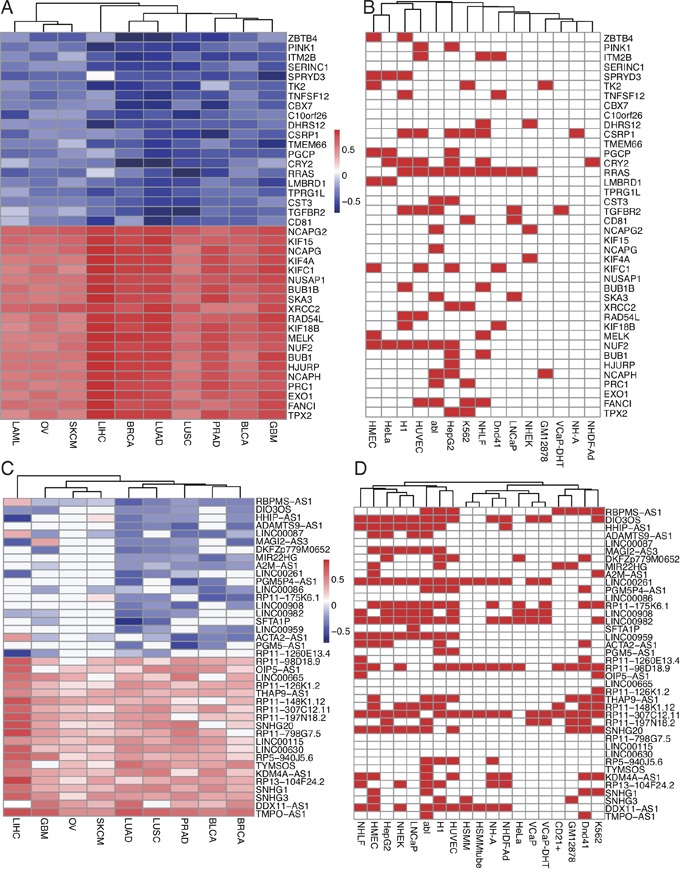
Co-expression analysis of EZH2 in TCGA data and comparison with ChIP sequencing results **A.** Heatmap of Spearman correlation coefficients between EZH2 and other mRNAs. Top ranked 20 positively and negatively correlated mRNAs were presented and compared with ChIP sequencing results. **B.** Red indicated that this mRNA gene was potential EZH2 target in the cell. **C.** Heatmap of Spearman correlation coefficients between EZH2 and lncRNAs. Top ranked 20 positively and negatively correlated lncRNAs were presented and compared with ChIP sequencing results. **D.** Red indicated that this lncRNA was potential EZH2 target in the cell.

For co-expressed mRNAs, the correlation coefficients between EZH2 and 19,697 coding genes were calculated by Spearman correlation test in ten tumor types (Supplementary Table S3) and was presented as heatmap in Supplementary Figure S2A. Genes with high coefficients were largely consistent among the ten tumor types, which is, EZH2 was either positively or negatively correlated with these genes in all ten tumors (Supplementary Figure S2A). The top ranked positively or negatively correlated 20 co-expressed genes were selected by calculating the row sum of the coefficients in ten tumors and were shown in Figure 5A. We also validated the correlation between the ChIP sequencing data and co-expressing data by checking whether the top 20 positively or negatively correlated co-expressed genes were the potential targets in the ChIP sequencing results (Figure 5B). Figure 5A and 5B showed that RRAS was negatively correlated with EZH2 in most tumors especially in LUSC, PRAD and LAML and was potential target in nine cell lines including abl, HepG2, K562 and LNCaP. TGFBR2, as another example, was negatively correlated with EZH2 in LUAD, LUSC and BRCA and was potential target in five cell lines including abl, LNCaP, and VCaP-DHT. NUF2 and PRC1 were positively correlated with EZH2 in TCGA data and were targets in six and two cell lines, respectively (Figure 5A and 5B). Binding of EZH2 at RRAS, TGFBR2, NUF2 and PRC1 loci in 19 cell lines were shown Supplementary Figure S3A.

For co-expressed lncRNAs, Co-expression analysis between EZH2 and lncRNAs were also applied using TCGA data from Co-LncRNA database. Spearman correlation coefficient in nine tumor types (LAML was not available) was obtained and NA values were represented as zero in the heatmap (Supplementary Table S4, Figure 5C and Supplementary Figure S2B). 1,401 lncRNAs with coefficients available at least in one tumor were shown in Supplementary Figure S2B, EZH2 co-expressed lncRNAs showed more diversity and cell specificity than mRNA, which is, some lncRNAs were positively correlated in some tumors while were negatively correlated in other tumors (Supplementary Figure S2B). Top ranked 20 positively and negatively correlated lncRNAs were shown in Figure 5C. We also checked whether these lncRNAs were potential targets in the ChIP sequencing data and results were shown in Figure 5D. Figure 5C and 5D showed that LINC00261 were negatively correlated with EZH2 in LUAD and LIHC and was potential target in 14 cell lines including H1, HMEC, HUVEC and NHLF cells. DIO3OS, another example, was negatively correlated with EZH2 in PRAD, BRCA and GBM and was potential target in 13 cell lines including K562, VCaP, VCaP-DHT, abl, HepG2 and NH-A (Figure 5C and 5D). RP11-307C12.11 and RP11-98D18.9 were another two examples which were positively correlated with EZH2 and were shown in Figure 5C and 5D. Binding of EZH2 at these four lncRNAs loci were shown in Supplementary Figure S3B.

## DISCUSSION

It is widely accepted that epigenetic alterations are associated with different stages of tumor formation and progression in many cancers. Therefore, epigenetic abnormalities in cancers are emerging as important biomarkers and may have therapeutic potential. As an integral component of PRC2, there are two possible molecular mechanisms for EZH2 action based on its role as a transcriptional repressor or activator [18, 20]. The canonical role of EZH2 is that of a histone methyltransferase which depends on PRC2 while the non-canonical role of EZH2 is less known as a transcriptional inducer which is independent to PRC2 [18].

Growing evidences have established that several essential genes are targeted by EZH2, ultimately leading to tumor growth or metastasis in several carcinomas especially in breast cancer and prostate cancer. In breast cancer, several lines of evidence have implicated overexpression of EZH2 and repression of tumor suppressors such as KRT5, KRT6, CDH3, RAD51 paralogs, CDKN1C, FOXC1, Wnt/β-catenin signaling (c-Myc, Cyclin D1, Axin2), RKIP and KLF2 [21]. Moreover, in prostate cancer, EZH2 can repress the expression of tumor suppressors such as DAB2IP, p16 (CDKN2A), CDK4, E-cadherin (CDH1), MSMB, Ras (KRAS), NF-κB (NFKB1), EMT regulator [21]. Similar findings have emerged in other human cancers including bladder cancer [22], melanoma [23], glioblastoma [24], liver [25], lung [26] and T-cell lymphoma carcinomas [27]. These aberrantly expressed genes above could increase in vitro and in vivo migration and metastasis or cause aberrant cell proliferation and tumor growth [18, 21].

These targets above have already been validated by functional experiments by different researchers, thus we tested if our bioinformatics analysis correctly predict these validated targets. Prostate cancer was chosen as an example since ChIP sequencing results were available in four prostate cancer cell lines in our data. An increased EZH2 expression in normal prostatic epithelial cells can suppress DAB2IP gene expression [28], DAB2IP was predicted as target in 14 cell lines including abl and LNCaP cell lines and was significantly correlated with EZH2 (Coef= -0.38, P<0.05) in RNA sequencing data in 497 prostate cancer patients (Supplementary Tables S1 and S3). Similarly, TIMP3 is suppressed in prostate cancer cells by EZH2 which promoted the degradation of the extracellular matrix (ECM) and metastasis [29], TIMP3 was a potential target in VCaP, VCaP-DHT, abl and LNCaP cell lines and negatively correlated with EZH2 (Coef= -0.52, P<0.05) in prostate cancer in our analysis (Supplementary Tables S1 and S3). In fact, CDKN2A, ADRB2, CDH1 [4, 30] and SLIT2 [31] were reported to be suppressed by EZH2 in prostate cancer cells and were also targeted by EZH2 in at least one prostate cell line in our ChIP sequencing analysis (Supplementary Table S1). These results proved that our bioinformatic predictions were reliable to a large extend and these validated EZH2 targets in prostate cancer might also be applied into other cell lines. It is worth noting that the correlations might not be compatible between the ChIP and RNA sequencing experiments results since they were conducted in different cells/tissues and under different conditions.

Interaction between EZH2 and lncRNAs in tumor also draws great attention to researchers in recent years. HOTAIR, one of the most well-known and studied lncRNA, was firstly found to recruit PRC2 in modulating the cancer epigenome in prostate cancer which depend on the enzymatic activity of EZH2 [32]. However, these studies were focused on how lncRNAs recruit EZH2 in re-targeting gene expression. Regulation of lncRNA expression by EZH2 remains largely unstudied. We systematically investigated the potential involvement of EZH2 in targeting lncRNA in 19 cell lines and revealed thousands of potential lncRNA targets for the first time.

Our study was an integrated ChIP sequencing and co-expression analysis study in 19 cell lines and ten cancer types, respectively. Moreover, this study was the first to reveal the potential lncRNA targets of EZH2 by ChIP sequencing. By integrating ChIP sequencing and co-expression analysis results, we revealed genome-wide shared and tissue/cell specific potential targets, researchers can predict potential targets in a specific cell line or cancer type and the underlying role of EZH2 in regulating as a suppressor or an activator. Thus, studies of EZH2 in tumorigenesis could be accelerated to understand the mechanism of EZH2, to identify novel markers for cancer diagnosis or treatment, and to prevent drug resistance in the future.

Given the evidence for EZH2 being a cancer driver, the development of EZH2-specific inhibitors has been an active area of investigation. Promising preclinical results have been obtained and human phase 1 trails are now underway with early results suggesting potential clinical activity [33-35]. Studies revealing the mechanisms of EZH2 in tumorigenesis may provide insights into the drug interaction or resistance in cancer treatment.

In conclusion, these divergent roles of EZH2 in human malignancies suggest context and tumor cell-type specificities. The contribution of hyperactive or hypoactive EZH2 during tumor formation may reflect the complex and important roles that EZH2-associated genes play in cell fate decisions [36]. Our study was the first to evaluate the role of EZH2 in regulating mRNA and lncRNA expression in tumor by integrating ChIP sequencing and co-expressing analysis. With more insights into the mechanisms of EZH2 in more cancer types, targeted therapy of EZH2 may apply to more cancer types and combination of other chemotherapies could be considered to overcome the drug resistance in clinical.

## MATERIALS AND METHODS

### ChIP sequencing data of EZH2 and RNA sequencing data in TCGA

For ChIP sequencing data, a comprehensive datasets search of Gene Expression Omnibus (GEO) database (http://www.ncbi.nlm.nih.gov/geo) was performed with search terms included “EZH2” combined with “ChIP”. To be eligible for inclusion in the study, ChIP sequencing datasets were selected under the following criteria: (1) antibody of EZH2 was used for chromatin enrichment; (2) IgG control was available; (3) high throughput sequencing rather than microarray was applied; (4) raw data was available. The ChIP sequencing data in 19 cell lines were obtained from GEO under the GSM number listed in Table 1. Cell line information was also listed. For RNA sequencing data, cancer types relevant with cell lines included in ChIP sequencing data were selected correspondingly. Level 3 RNA sequencing data were downloaded from The Cancer Genome Atlas (TCGA) Research Network (http://cancergenome.nih.gov/) using FireBrowseR [37]. Ten cancer types and sample numbers were listed in Table 2.

**Table 2:** RNA sequencing data from TCGA used in this study

Cancer types	Abbreviation	Sample size
Bladder urothelial carcinoma	BLCA	408
Breast invasive carcinoma	BRCA	1093
Glioblastoma multiforme	GBM	160
Acute Myeloid Leukemia	LAML	179
Liver hepatocellular carcinoma	LIHC	371
Lung adenocarcinoma	LUAD	515
Lung squamous cell carcinoma	LUSC	501
Ovarian serous cystadenocarcinoma	OV	304
Prostate adenocarcinoma	PRAD	497
Skin Cutaneous Melanoma	SKCM	469

### EZH2 binding peaks calling

Mapping of reads was done using bowtie (version 1.1.2) [38] on the hg19 genome. Bowtie's output was converted to BAM format using SAMtools (version 1.4) [39]. MACS2 (version 2.0.10) [40] was used to identify enriched ChIP regions of EZH2, and peak calling was employed to infer the actual binding sites from the positional distribution of sequenced DNA fragments. The q value (minimum FDR) cutoff to call significant regions was 0.01 as default setting.

### Potential target gene annotations, GO and KEGG pathway analysis

Bioconductor package ChIPseeker (version 1.6.7) [41] within R program (version 3.2.4) was facilitated to detect annotation of the enriched peaks of EZH2 binding sites. For potential mRNA targets, binding peaks were annotated by “TxDb.Hsapiens.UCSC.hg19.knownGene” package while for potential lncRNA targets, binding peaks were annotated by long noncoding RNA GTF file downloaded from GENECODE database (http://www.gencodegenes.org/) (Release 24). Transcription start site (TSS) region, by default TSS is defined from -3kb to +3kb.

Gene Ontology (GO) enrichment and Kyoto Encyclopedia of Genes and Genomes (KEGG) pathway analysis were conducted using clusterProfiler package (version 2.4.3) [42]. Annotated peaks were further analyzed for differences of dynamic variation of EZH2 binding sites using ChIPseeker.

### Heatmap of ChIP sequencing signal of EZH2 binding sites

SeqMINER-1.3.3e, an integrated user-friendly platform that allows addressing central questions in the ChIP sequencing analysis work flow [43], was used to generate the heatmap of ChIP sequencing signal of EZH2 binding sites and conduct K-means clustering analysis. This fine-scale analysis highlights differential binding sites of individual TSSs and flanking regions, as well as differential relations to gene activity.

### Co-expression analysis of EZH2 in TCGA data

Level 3 mRNA sequencing data were downloaded from TCGA and Spearman correlation test was preformed between the RSEM of EZH2 and other coding genes. Co-expression analysis between EZH2 and lncRNAs were also applied using TCGA data from Co-LncRNA database (http://www.bio-bigdata.com/Co-LncRNA/). Spearman correlation coefficients in nine tumor types (LAML was not available) were obtained, *NA* values were detected and replaced as zero due to the relatively low expression and cell specificity of lncRNAs in different cancer types. Top ranked co-expressed mRNAs and lncRNAs were defined as the maximum of row sum of coefficients in ten or nine cancer types, respectively. Heatmaps of coefficients between EZH2 and mRNAs and lncRNAs were also generated, respectively.

## SUPPLEMENTARY MATERIALS











## References

[1] Orkin SH, Hochedlinger K (2011). Chromatin connections to pluripotency and cellular reprogramming. Cell.

[2] Rada-Iglesias A, Bajpai R, Swigut T, Brugmann SA, Flynn RA, Wysocka J (2011). A unique chromatin signature uncovers early developmental enhancers in humans. Nature.

[3] Margueron R, Reinberg D (2011). The Polycomb complex PRC2 and its mark in life. Nature.

[4] Varambally S, Dhanasekaran SM, Zhou M, Barrette TR, Kumar-Sinha C, Sanda MG, Ghosh D, Pienta KJ, Sewalt RG, Otte AP, Rubin MA, Chinnaiyan AM (2002). The polycomb group protein EZH2 is involved in progression of prostate cancer. Nature.

[5] Bracken AP, Pasini D, Capra M, Prosperini E, Colli E, Helin K (2003). EZH2 is downstream of the pRB-E2F pathway, essential for proliferation and amplified in cancer. EMBO J.

[6] Bachmann IM, Halvorsen OJ, Collett K, Stefansson IM, Straume O, Haukaas SA, Salvesen HB, Otte AP, Akslen LA (2006). EZH2 expression is associated with high proliferation rate and aggressive tumor subgroups in cutaneous melanoma and cancers of the endometrium, prostate, and breast. J Clin Oncol.

[7] Sauvageau M, Sauvageau G (2010). Polycomb group proteins: multi-faceted regulators of somatic stem cells and cancer. Cell Stem Cell.

[8] Cao Q, Yu J, Dhanasekaran SM, Kim JH, Mani RS, Tomlins SA, Mehra R, Laxman B, Cao X, Yu J, Kleer CG, Varambally S, Chinnaiyan AM (2008). Repression of E-cadherin by the polycomb group protein EZH2 in cancer. Oncogene.

[9] Fujii S, Ito K, Ito Y, Ochiai A (2008). Enhancer of zeste homologue 2 (EZH2) down-regulates RUNX3 by increasing histone H3 methylation. J Biol Chem.

[10] Shi B, Liang J, Yang X, Wang Y, Zhao Y, Wu H, Sun L, Zhang Y, Chen Y, Li R, Zhang Y, Hong M, Shang Y (2007). Integration of estrogen and Wnt signaling circuits by the polycomb group protein EZH2 in breast cancer cells. Mol Cell Biol.

[11] Rinn JL, Chang HY (2012). Genome regulation by long noncoding RNAs. Annu Rev Biochem.

[12] Hirata H, Hinoda Y, Shahryari V, Deng G, Nakajima K, Tabatabai ZL, Ishii N, Dahiya R (2015). Long Noncoding RNA MALAT1 Promotes Aggressive Renal Cell Carcinoma through Ezh2 and Interacts with miR-205. Cancer research.

[13] Jia J, Li F, Tang XS, Xu S, Gao Y, Shi Q, Guo W, Wang X, He D, Guo P (2016). Long noncoding RNA DANCR promotes invasion of prostate cancer through epigenetically silencing expression of TIMP2/3. Oncotarget.

[14] Wan L, Sun M, Liu GJ, Wei CC, Zhang EB, Kong R, Xu TP, Huang MD, Wang ZX (2016). Long Noncoding RNA PVT1 Promotes Non-Small Cell Lung Cancer Cell Proliferation through Epigenetically Regulating LATS2 Expression. Mol Cancer Ther.

[15] Zhu P, Wang Y, Huang G, Ye B, Liu B, Wu J, Du Y, He L, Fan Z (2016). lnc-beta-Catm elicits EZH2-dependent beta-catenin stabilization and sustains liver CSC self-renewal. Nat Struct Mol Biol.

[16] Sun M, Liu XH, Lu KH, Nie FQ, Xia R, Kong R, Yang JS, Xu TP, Liu YW, Zou YF, Lu BB, Yin R, Zhang EB, Xu L, De W, Wang ZX (2014). EZH2-mediated epigenetic suppression of long noncoding RNA SPRY4-IT1 promotes NSCLC cell proliferation and metastasis by affecting the epithelial-mesenchymal transition. Cell Death Dis.

[17] Sun Q, Liu H, Li L, Zhang S, Liu K, Liu Y, Yang C (2015). Long noncoding RNA-LET, which is repressed by EZH2, inhibits cell proliferation and induces apoptosis of nasopharyngeal carcinoma cell. Med Oncol.

[18] Deb G, Thakur VS, Gupta S (2013). Multifaceted role of EZH2 in breast and prostate tumorigenesis: epigenetics and beyond. Epigenetics.

[19] Tang B, Hsu PY, Huang TH, Jin VX (2013). Cancer omics: from regulatory networks to clinical outcomes. Cancer Lett.

[20] Bae WK, Hennighausen L (2014). Canonical and non-canonical roles of the histone methyltransferase EZH2 in mammary development and cancer. Mol Cell Endocrinol.

[21] Kim KH, Roberts CW (2016). Targeting EZH2 in cancer. Nature medicine.

[22] Raman JD, Mongan NP, Tickoo SK, Boorjian SA, Scherr DS, Gudas LJ (2005). Increased expression of the polycomb group gene, EZH2, in transitional cell carcinoma of the bladder. Clin Cancer Res.

[23] Zingg D, Debbache J, Schaefer SM, Tuncer E, Frommel SC, Cheng P, Arenas-Ramirez N, Haeusel J, Zhang Y, Bonalli M, McCabe MT, Creasy CL, Levesque MP, Boyman O, Santoro R, Shakhova O (2015). The epigenetic modifier EZH2 controls melanoma growth and metastasis through silencing of distinct tumour suppressors. Nat Commun.

[24] Lee J, Son MJ, Woolard K, Donin NM, Li A, Cheng CH, Kotliarova S, Kotliarov Y, Walling J, Ahn S, Kim M, Totonchy M, Cusack T, Ene C, Ma H, Su Q (2008). Epigenetic-mediated dysfunction of the bone morphogenetic protein pathway inhibits differentiation of glioblastoma-initiating cells. Cancer Cell.

[25] Sudo T, Utsunomiya T, Mimori K, Nagahara H, Ogawa K, Inoue H, Wakiyama S, Fujita H, Shirouzu K, Mori M (2005). Clinicopathological significance of EZH2 mRNA expression in patients with hepatocellular carcinoma. Br J Cancer.

[26] Hussain M, Rao M, Humphries AE, Hong JA, Liu F, Yang M, Caragacianu D, Schrump DS (2009). Tobacco smoke induces polycomb-mediated repression of Dickkopf-1 in lung cancer cells. Cancer research.

[27] Yan J, Ng SB, Tay JL, Lin B, Koh TL, Tan J, Selvarajan V, Liu SC, Bi C, Wang S, Choo SN, Shimizu N, Huang G, Yu Q, Chng WJ (2013). EZH2 overexpression in natural killer/T-cell lymphoma confers growth advantage independently of histone methyltransferase activity. Blood.

[28] Chen H, Tu SW, Hsieh JT (2005). Down-regulation of human DAB2IP gene expression mediated by polycomb Ezh2 complex and histone deacetylase in prostate cancer. J Biol Chem.

[29] Shin YJ, Kim JH (2012). The role of EZH2 in the regulation of the activity of matrix metalloproteinases in prostate cancer cells. PLoS One.

[30] Tamgue O, Chai CS, Hao L, Zambe JC, Huang WW, Zhang B, Lei M, Wei YM (2013). Triptolide inhibits histone methyltransferase EZH2 and modulates the expression of its target genes in prostate cancer cells. Asian Pac J Cancer Prev.

[31] Yu J, Cao Q, Yu J, Wu L, Dallol A, Li J, Chen G, Grasso C, Cao X, Lonigro RJ, Varambally S, Mehra R, Palanisamy N, Wu JY, Latif F, Chinnaiyan AM (2010). The neuronal repellent SLIT2 is a target for repression by EZH2 in prostate cancer. Oncogene.

[32] Gupta RA, Shah N, Wang KC, Kim J, Horlings HM, Wong DJ, Tsai MC, Hung T, Argani P, Rinn JL, Wang Y, Brzoska P, Kong B, Li R, West RB, van de Vijver MJ (2010). Long non-coding RNA HOTAIR reprograms chromatin state to promote cancer metastasis. Nature.

[33] Knutson SK, Wigle TJ, Warholic NM, Sneeringer CJ, Allain CJ, Klaus CR, Sacks JD, Raimondi A, Majer CR, Song J, Scott MP, Jin L, Smith JJ, Olhava EJ, Chesworth R, Moyer MP (2012). A selective inhibitor of EZH2 blocks H3K27 methylation and kills mutant lymphoma cells. Nat Chem Biol.

[34] McCabe MT, Ott HM, Ganji G, Korenchuk S, Thompson C, Van Aller GS, Liu Y, Graves AP, Della Pietra A, Diaz E, LaFrance LV, Mellinger M, Duquenne C, Tian X, Kruger RG, McHugh CF (2012). EZH2 inhibition as a therapeutic strategy for lymphoma with EZH2-activating mutations. Nature.

[35] Knutson SK, Kawano S, Minoshima Y, Warholic NM, Huang KC, Xiao Y, Kadowaki T, Uesugi M, Kuznetsov G, Kumar N, Wigle TJ, Klaus CR, Allain CJ, Raimondi A, Waters NJ, Smith JJ (2014). Selective inhibition of EZH2 by EPZ-6438 leads to potent antitumor activity in EZH2-mutant non-Hodgkin lymphoma. Mol Cancer Ther.

[36] Kondo Y (2014). Targeting histone methyltransferase EZH2 as cancer treatment. J Biochem.

[37] Rahman M, Jackson LK, Johnson WE, Li DY, Bild AH, Piccolo SR (2015). Alternative preprocessing of RNA-Sequencing data in The Cancer Genome Atlas leads to improved analysis results. Bioinformatics.

[38] Langmead B, Trapnell C, Pop M, Salzberg SL (2009). Ultrafast and memory-efficient alignment of short DNA sequences to the human genome. Genome Biol.

[39] Li H, Handsaker B, Wysoker A, Fennell T, Ruan J, Homer N, Marth G, Abecasis G, Durbin R, Genome Project Data Processing S (2009). The Sequence Alignment/Map format and SAMtools. Bioinformatics.

[40] Zhang Y, Liu T, Meyer CA, Eeckhoute J, Johnson DS, Bernstein BE, Nusbaum C, Myers RM, Brown M, Li W, Liu XS (2008). Model-based analysis of ChIP-Seq (MACS). Genome Biol.

[41] Yu G, Wang LG, He QY (2015). ChIPseeker: an R/Bioconductor package for ChIP peak annotation, comparison and visualization. Bioinformatics.

[42] Yu G, Wang LG, Han Y, He QY (2012). clusterProfiler: an R package for comparing biological themes among gene clusters. OMICS.

[43] Ye T, Krebs AR, Choukrallah MA, Keime C, Plewniak F, Davidson I, Tora L (2011). seqMINER: an integrated ChIP-seq data interpretation platform. Nucleic Acids Res.

[44] Chng KR, Chang CW, Tan SK, Yang C, Hong SZ, Sng NY, Cheung E (2012). A transcriptional repressor co-regulatory network governing androgen response in prostate cancers. EMBO J.

[45] Ram O, Goren A, Amit I, Shoresh N, Yosef N, Ernst J, Kellis M, Gymrek M, Issner R, Coyne M, Durham T, Zhang X, Donaghey J, Epstein CB, Regev A, Bernstein BE (2011). Combinatorial patterning of chromatin regulators uncovered by genome-wide location analysis in human cells. Cell.

[46] Xu K, Wu ZJ, Groner AC, He HH, Cai C, Lis RT, Wu X, Stack EC, Loda M, Liu T, Xu H, Cato L, Thornton JE, Gregory RI, Morrissey C, Vessella RL (2012). EZH2 oncogenic activity in castration-resistant prostate cancer cells is Polycomb-independent. Science.

