# Supplementary material for: Integrated bioinformatics analysis of chromatin regulator EZH2 in regulating mRNA and lncRNA expression by ChIP sequencing and RNA sequencing
Source: Oncotarget. 2016 Nov 7;7(49):81715–26. doi: 10.18632/oncotarget.13169 (PMC5348424; doi:10.18632/oncotarget.13169)
Supplement: Supplementary file 1 [file oncotarget-07-81715-s001.pdf]

Integrated bioinformatics analysis of chromatin regulator EZH2 in regulating mRNA and lncRNA expression by ChIP sequencing and RNA sequencing

SUPPLEMENTARY MATERIALS

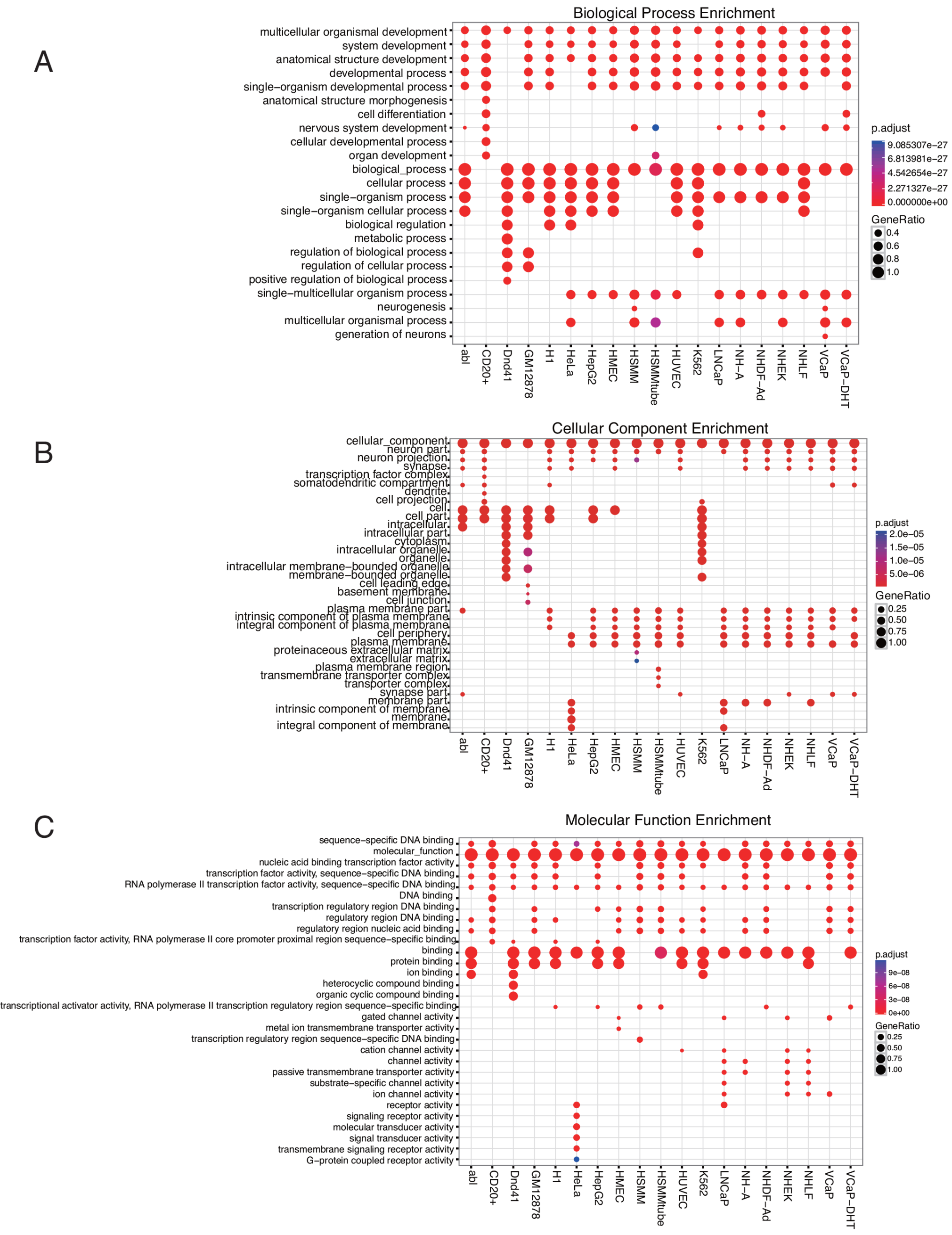

Supplementary Figure S1: GO enrichment analysis of EZH2 target genes in 19 cell lines. A. Biological process enrichment analysis of EZH2 target genes. **B.** Cellular component enrichment analysis of EZH2 target genes. **C.** Molecular function enrichment of EZH2 target genes.

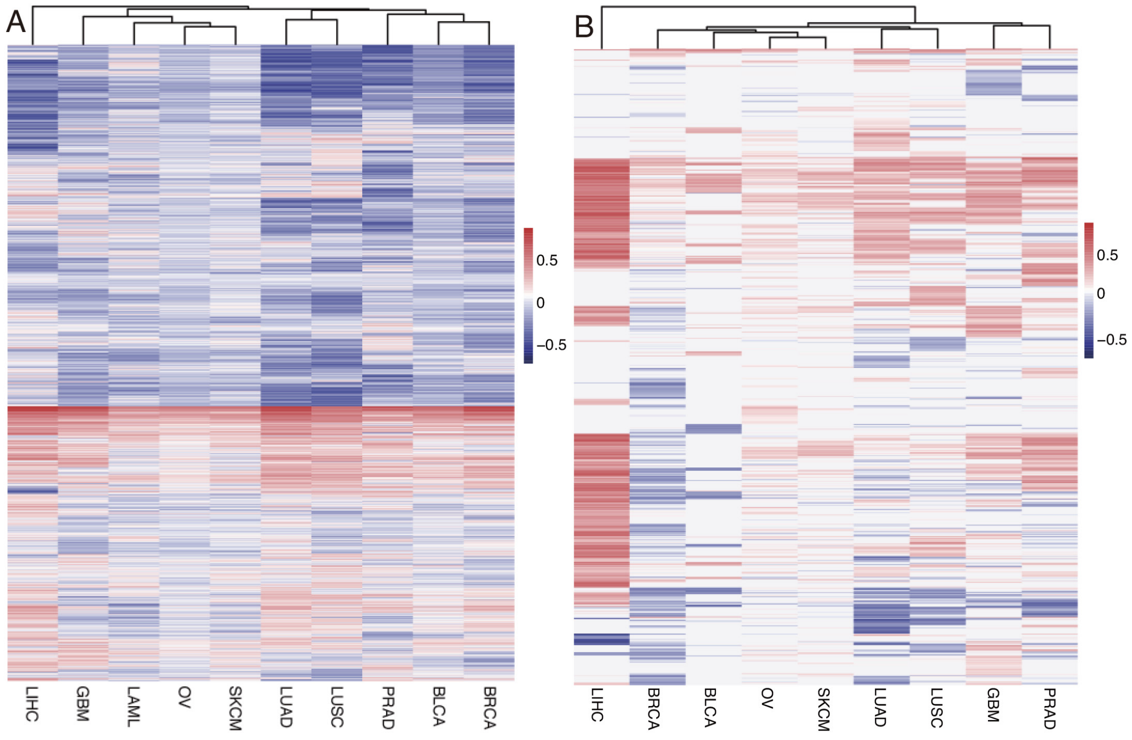

Supplementary Figure S2: Heatmap of the Spearman correlation coefficients between EZH2 and mRNAs or lncRNAs. **A.** Heatmap of the Spearman correlation coefficients between EZH2 and mRNAs. A total of 19,679 coding genes was presented. **B.** Heatmap of the Spearman correlation coefficients between EZH2 and lncRNAs. 1,401 lncRNAs with coefficients available at least in one tumor were shown.

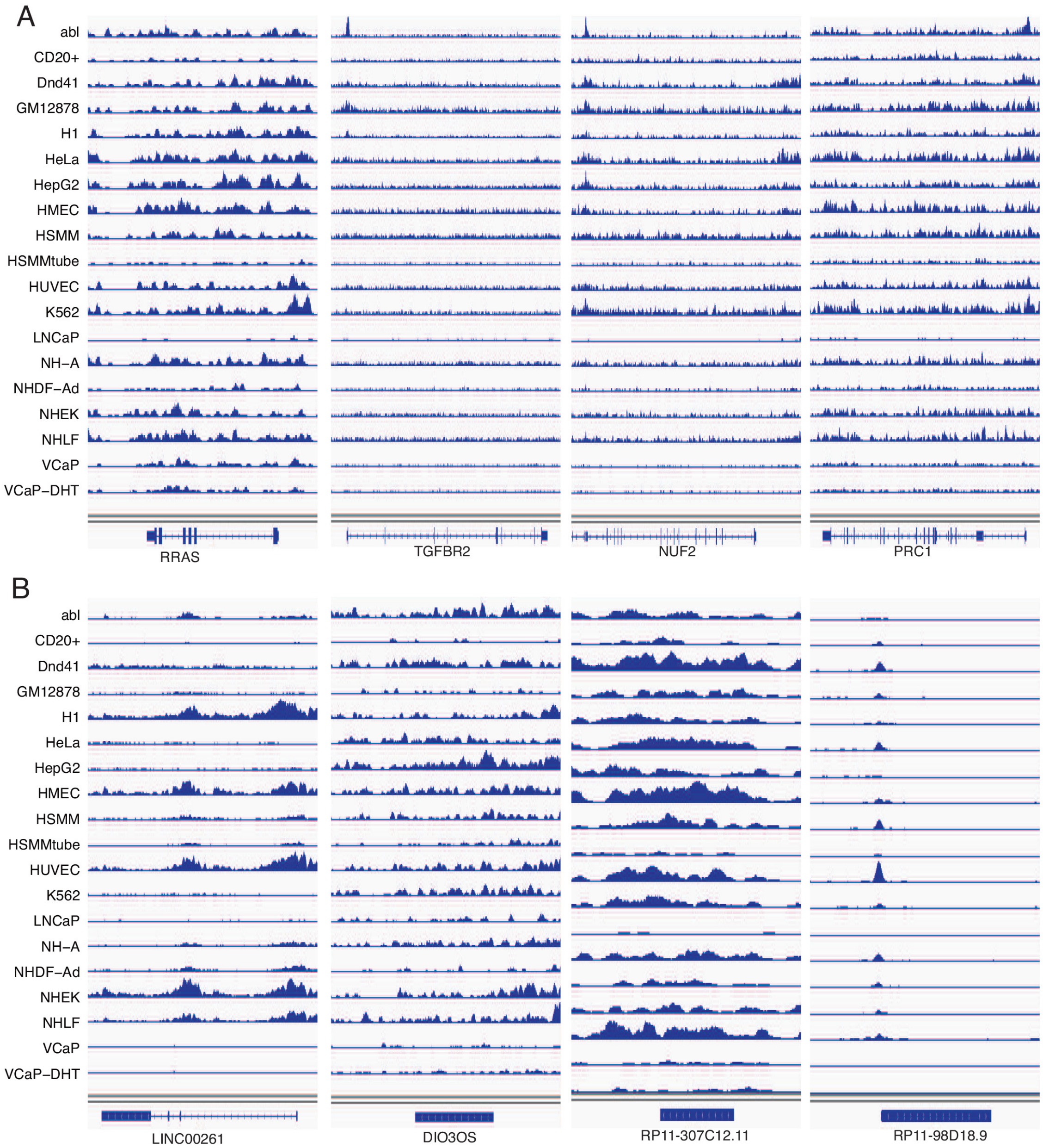

Supplementary Figure S3: Binding of EZH2 at representative genomic loci. A. Binding of EZH2 at four target coding genes including RRAS, TGFBR2, NUF2 and PRC1 were shown in 19 cell lines. **B.** Binding of EZH2 at four target lncRNAs including LINC00261, DIO3OS, RP11-307C12.11 and RP11098D18.9 were shown in 19 cell lines.

Supplementary Table S1: Potential mRNA targets of EZH2 in 19 cell lines

See Supplementary File 1

Supplementary Table S2: Potential lncRNA targets of EZH2 in 19 cell lines

See Supplementary File 2

Supplementary Table S3: Coefficient between EZH2 and mRNAs in TCGA

See Supplementary File 3

Supplementary Table S4: Coefficient between EZH2 and lncRNAs in TCGA

See Supplementary File 4
